# Etiology and Risk Factors for Shunt Revision in Adult Hydrocephalus: A Single-Center Retrospective Cohort Study

**DOI:** 10.3390/brainsci16030318

**Published:** 2026-03-17

**Authors:** Christodoulos Komiotis, Anastasia Tasiou, Alexandros G. Brotis, Kostas N. Fountas

**Affiliations:** 1 Department of Neurosurgery, University Hospital of Larissa, 41110 Larissa, Greece; xkomiotis@gmail.com (C.K.); ttasiou@yahoo.com (A.T.); alexgbrodis@yahoo.com (A.G.B.); 2 Faculty of Medicine, School of Health Sciences, University of Thessaly, 41110 Larissa, Greece

**Keywords:** adults, cerebrospinal fluid, hydrocephalus, revision rate, shunt failure, ventriculoperitoneal shunt

## Abstract

**Highlights:**

**What are the main findings?**

• This study highlights the necessity for revision in CSF shunting procedures.
• Infection constitutes the most common reason for shunt revision.

**What are the implications of the main findings?**

• In our cohort, the revision-free survival was 86.4% at 12 months.
• Patient age is associated with shunt failure and subsequent revision.

**Abstract:**

**Background/Objectives**: Hydrocephalus is defined as the symptomatic accumulation of excessive cerebrospinal fluid (CSF) within the ventricular system. It has an estimated incidence of 85 cases per 100,000 population annually in adults, making it one of the most common conditions managed by neurosurgeons globally. Many conditions may lead to ventricular dilation and hydrocephalus, such as hemorrhage, tumors, infection, trauma, and idiopathic normal-pressure hydrocephalus (iNPH). Regardless of the cause, the gold-standard treatment for hydrocephalus is CSF diversion, usually via a ventriculoperitoneal (VP) shunt. The goal of the present study is to present our experience regarding the etiology of hydrocephalus, management, and shunt failure characteristics over the last 11 years. **Methods**: A single-center retrospective cohort study was performed. Our cohort consisted of adult patients who were shunted or required revision surgery in our department over the last 11 years. Data regarding the etiology of hydrocephalus, management, shunt characteristics, revision status, and etiology of revision were collected and retrospectively analyzed. Univariable and multivariable logistic regression models were established in order to explore potential associations between the etiology of hydrocephalus and patient characteristics and risk of shunt revision. Revision-free survival probabilities were estimated using the Kaplan–Meier method, while shunt failure rates were also calculated. **Results**: Our cohort consisted of 114 patients, the median age was 59 (IQR = 26.5) years, and the male-to-female ratio was 1.04:1. The most common cause of hydrocephalus was iNPH (30.7%), followed by post-hemorrhagic (23.7%) and tumor-related hydrocephalus (21.1%). The 12-month revision rate was 13.6%, with overall revision-free survival of 86.4% at one year. Infection (43.2%) was the most common cause of shunt revision, followed by obstruction (16.2%), and mechanical disconnection and migration (18.9%). Younger age was associated with higher risk of revision, while etiology of hydrocephalus and patient sex were not. **Conclusions**: Our study adds to the pertinent literature data regarding hydrocephalus etiology, management strategies, and shunt failure rates across different hydrocephalus etiologies. Additionally, it serves as a foundation for future studies that could identify predictors of shunt failure, apart from the etiology of hydrocephalus, such as patient characteristics, surgical factors, or shunt types. Finally, we highlight the importance of comprehensive national and potentially continental registries, which will facilitate large-scale analyses.

## 1. Introduction

Hydrocephalus is defined as the symptomatic accumulation of excessive cerebrospinal fluid (CSF) within the ventricular system [1,2]. In adults, its estimated incidence is 85 cases per 100,000 population annually, making it one of the most common neurosurgical conditions worldwide [3,4]. CSF is mainly produced by the choroid plexi within the lateral, third and fourth ventricles, at an approximate rate of 500 mL per day. It circulates through the ventricular system, exits to the subarachnoid space via the foramina of Magendie and Luschka, and is eventually absorbed into the venous system via the arachnoid granulations [1,2,5]. Disruption in CSF production, flow, or absorption may result in active dilatation of the ventricular system and clinical symptomatology, known as hydrocephalus.

Since it was first described back in 1913 [6], many classifications have been proposed, including communicating and non-communicating and, in adults, obstructive, communicating, hypersecretory, and idiopathic normal-pressure hydrocephalus (iNPH) [7]. More specifically, adult hydrocephalus can be further subdivided, based on the underlying pathophysiology, into congenital (becomes symptomatic in adulthood), post-hemorrhagic, post-infectious, post-traumatic, tumor-related, and iNPH [7,8]. Regardless of its etiology, hydrocephalus may lead to serious symptoms, including headache, neck pain, nausea, vomiting, lethargy, blurred vision, diplopia, seizures, and even coma and death. iNPH may present with the classic triad or any of cognitive decline, gait impairment, and urinary incontinence [7,9,10,11]. Therefore, the overall outcome for these patients is associated with the timely and appropriate management of their disease. CSF diversion procedures, such as ventriculoperitoneal (VP) and, less commonly, ventriculoatrial (VA) or lumboperitoneal (LP) shunts, remain the cornerstone of treatment for hydrocephalus. In several cases, endoscopic third ventriculostomy (ETV) could be an alternative treatment option, mainly for patients with identified structural flow blockage [12]. Despite the linearly increasing number of performed ETV procedures, shunting remains the most effective treatment currently available. Shunt-related complications that may lead to shunt malfunction and the patient’s clinical deterioration include intracerebral or intraventricular hemorrhage, infection, mechanical blockage, shunt malposition, disconnection or migration, and over- or under-drainage, as well as subdural hematomas and seizures [12,13,14,15,16,17,18,19].

To our knowledge, data on shunt failure rates across different hydrocephalus etiologies and their subcategories remain contradictory. In our single-center, retrospective cohort study, we aim to describe the underlying hydrocephalus etiologies in adult patients treated in our institution over the past 11 years, and report the management strategies, as well as any treatment-related complications. We also aim to evaluate 12-month diversion durability by estimating revision-free survival in a cohort of patients undergoing shunt insertion for various etiologies, comparing revision-free survival between iNPH and secondary hydrocephalus due to other etiologies and identifying predictors of shunt revision using univariate and multivariable logistic regression modeling. Finally, a nomogram was constructed based on the final multivariate model to enhance the applicability of our results by estimating individualized risk of revision.

## 2. Materials and Methods

### 2.1. Study Design and Population

We conducted a retrospective, single-center cohort study of adult patients (≥16 years) who underwent surgical management for hydrocephalus at the University Hospital of Larissa over an 11-year period (2015–2025). Patients were identified through the institutional neurosurgical records. Our patients were divided into groups: (i) patients who underwent initial shunting and any subsequent necessary revision in our institution during the study period (Group A), and (ii) patients who underwent shunt revision at our institution, while having undergone initial shunt insertion either in another institution or in our department before the current study period (Group B). Group A was our main study cohort, whereas Group B was only used for descriptive statistics of hydrocephalus etiology and shunt revision.

### 2.2. Data Collection

Data were extracted from hospital and operative records. Our study was approved by our Institutional Review Board and performed according to the Declaration of Helsinki and Health Insurance Portability and Accountability Act (HIPAA).

Variables such as age at the time of surgery, sex, type of surgery (VP shunt, VA shunt, ETV), and cause of hydrocephalus, classified as idiopathic (iNPH), congenital and secondary, for example post-hemorrhagic [including aneurysmal subarachnoid hemorrhage (aSAH), arteriovenous malformation (AVM), or cavernous malformation (CM) rupture], post-infectious, tumor-related, or post-traumatic, were collected. Several patients had more than one factor responsible for hydrocephalus development, for example hemorrhage and postoperative meningitis. Such patients were grouped into one category, according to what the triggering factor that led to hydrocephalus was, or what the factor that most likely led to hydrocephalus was, based on clinical and imaging criteria. In post-infectious hydrocephalus cases, the organism responsible for the infection was recorded when available, while patients with tumor-related hydrocephalus were grouped according to the benign or malignant nature of the causative tumors. Additionally, the anatomic location of obstruction, the shunt type, and the initial valve opening pressure in mmH_2_O were recorded. Patients treated by ETV were included in our descriptive statistics of hydrocephalus etiology and management; however, they were excluded from our shunt failure analysis. Patients underwent regular follow-up for 12 months, with additional assessments performed in the event of clinical deterioration.

**Figure 1:**
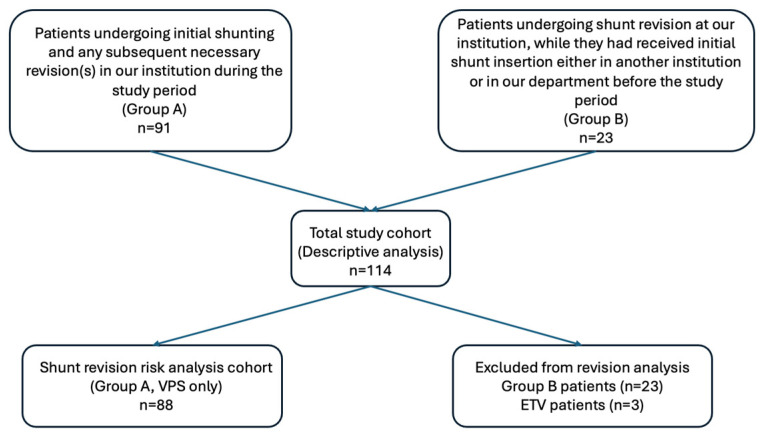
Schematic depiction of cohort selection process.

Τhe cause of revision was recorded in all patients, while the time interval from initial shunting to revision was recorded only for patients in Group A. The cause of revision was identified as obstruction of the shunt (proximal or distal), shunt malfunction, disconnection and/or migration of either the proximal or distal catheter, or infection (divided into draining site or CSF infection). The underlying pathogen organism was recorded in those cases that could be isolated. Our cohort selection process is summarized in Figure 1.

### 2.3. Statistical Analysis

Descriptive statistics were used to summarize patient demographics and clinical characteristics. Categorical variables were reported as frequencies and percentages, while continuous variables were reported as medians, ranges, and interquartile ranges (IQRs).

Subgroup analyses were conducted to further characterize hydrocephalus etiology within broader categories. Specifically, the post-hemorrhagic group was subdivided into aneurysm-related, AVM-related, and CM-related hydrocephalus, while the tumor-related group was categorized into malignant and benign subtypes. When available, histological data were reported for tumor-related cases, and causative organisms were documented for post-infectious hydrocephalus.

The revision rate was calculated among patients who initially underwent shunting in our institution (Group A). For descriptive purposes, all patients who underwent revision, regardless of the institution at which the first shunting took place, were included to characterize the cause and the type of revision. Due to the limited number of revision cases, statistical analysis was primarily descriptive.

Baseline characteristics for the specific revision analysis sub-cohort were summarized according to revision status. Here, continuous variables are presented as the mean ± standard deviation (SD), and categorical variables as frequencies and percentages. Time-to-event analysis was performed to evaluate shunt durability over 12 months. Shunt failure was defined as the need for surgical revision. Revision-free survival probabilities were estimated using the Kaplan–Meier method and reported at 1, 3, 6, 9, and 12 months. Median revision-free survival was calculated whenever reached. Survival curves were compared between iNPH and secondary hydrocephalus using the log-rank test. To identify predictors of revision within 12 months, univariate logistic regression analyses were first performed. Variables of clinical relevance (age, sex, and classification) were subsequently entered into a multivariate logistic regression model. Odds ratios (ORs) with 95% confidence intervals (CIs) were reported. Model discrimination was assessed using the c-statistic (area under the receiver operating characteristic curve), and calibration was evaluated using the Hosmer–Lemeshow goodness-of-fit test. A nomogram was constructed from the multivariate model to provide individualized risk prediction. Statistical significance was defined as a two-sided *p*-value < 0.05. All statistical analyses were carried out using the statistical environment R.

## 3. Results

### 3.1. Patient Characteristics

Overall, 114 patients were included in this retrospective cohort study. In total, 145 operations were performed, including 88 new insertions of VP shunts or other permanent CSF diversion operations, 3 ETVs, and 54 revision cases. There were 91 (79.8%) patients belonging to Group A, while 23 (20.2%) belonged to Group B.

Our study population included 58 (50.9%) males and 56 (49.1%) females, with a male-to-female ratio of approximately 1.04:1. The median age was 59 years (IQR = 26.5, range = 16–81). Demographic data are summarized in Table 1. There was only one patient who died from shunt failure.

### 3.2. Etiology of Hydrocephalus

#### 3.2.1. Overall Etiology Distribution

The most common underlying cause of hydrocephalus in our cohort was secondary causes, followed by iNPH. More specifically, 35 (30.7%) patients suffered from iNPH, 27 (23.7%) from post-hemorrhagic hydrocephalus, and 24 (21.1%) from tumor-related hydrocephalus. Additionally, post-traumatic and congenital hydrocephalus was identified as an underlying cause in 10 (8.8%), and 8 (7%) patients, respectively. Furthermore, six patients (5.3%) developed post-infectious hydrocephalus, while four (3.5%) had hydrocephalus due to various reasons, such as acquired aqueduct stenosis in three patients, and post-radiation hydrocephalus in one patient.

#### 3.2.2. Secondary Hydrocephalus Subgroups

(a) Congenital hydrocephalus

Eight patients (three males and five females) with congenital hydrocephalus were included in our cohort. Among them, three had congenital aqueduct stenosis, while three had Chiari malformation (one of them also had a myelomeningocele). Data were not available for the other two cases.

(b) Tumor-related hydrocephalus

Twenty-four patients (13 males and 11 females) with tumor-related hydrocephalus were included in this study. Overall, 11 patients suffered from malignant tumors, three patients with glioma, three with metastases, two with medulloblastoma, and one with central nervous system (CNS) lymphoma, while in two cases, histological diagnosis was not available. Eleven patients had benign tumors: three patients with meningioma, two with craniopharyngioma, one with pilocytic astrocytoma, one with giant cell astrocytoma, one with vestibular schwannoma, two with central neurocytoma, and one with pineocytoma. Another patient had obstructive hydrocephalus due to a pineal lesion, and another one had a radiologically suspected lymphoma, but no biopsy had been obtained.

(c) Post-hemorrhagic hydrocephalus

There were 27 patients (12 males and 15 females) with post-hemorrhagic hydrocephalus. The most common cause of hemorrhage leading to hydrocephalus was aSAH, occurring in 17 patients. Hemorrhage due to an AVM rupture occurred in two patients, while three more developed hydrocephalus after a CM hemorrhage. Furthermore, two patients had hydrocephalus due to a tumor-associated hemorrhage, and another one developed hydrocephalus due to previous germinal matrix hemorrhage. In one patient, the cause of hemorrhage remained unknown.

(d) Post-infectious hydrocephalus

There were six patients (three males and three females) with post-infectious hydrocephalus. Among them, two had meningitis, one had a brain abscess, and another one had a postoperative CNS infection.

### 3.3. Anatomical Area of CSF Obstruction

Regarding the area of CSF obstruction, the subarachnoid space was the most common anatomic location (83 patients, 72.8%), followed by the cerebral aqueduct (15 patients, 13.2%), and the foramina of Monroe (five patients, 4.4%). The fourth and third ventricles were the areas of blockage for six (5.3%) and four (3.5%) patients, respectively. Finally, the area of obstruction was not recorded in one (0.9%) patient. The etiology and the area of obstruction are shown in Table 1.

### 3.4. Hydrocephalus Management and Shunt Characteristics

Overall, the vast majority of our patients (110/114, 96.5%) were managed by a VP shunt. One of them required bilateral shunting, so in total, 111 VP shunt systems were inserted. One patient (0.9%) received a VA shunt, while three patients (2.6%) underwent ETV. A free-hand insertion technique was performed in all patients who underwent first VP shunt insertion in our institution.

Shunt type and manufacturer information were available for 107/112 shunt systems. The Codman Hakim programmable shunt (Integra LifeSciences, Princeton, NJ, USA) was the most commonly used, as it was the preferred system in 102/112 (91.1%) of cases. The Sphera Pro programmable shunt (PMH—Produtos Médicos Hospitalares, S.A, Samora, Correia, Portugal) and the Polaris programmable shunt (Sophysa, Orsay, France) were used in two (1.7%) patients each. In the patient that was treated with a VA shunt, the Integra OSV II valve with an anti-siphon mechanism (Integra LifeSciences, Princeton, NJ, USA) was implanted (0.9%). Shunt type and manufacturer remained unknown for five (4.5%) systems.

Initial valve opening pressure settings ranged from 90 to 160 mmH_2_O. For classification purposes, values were grouped into three categories: ≤110 mmH_2_O, 120–130 mmH_2_O, and ≥140 mmH_2_O. Among the 100 patients with available data, the majority (78%) had valves set at pressures between 120 mmH_2_O and 130 mmH_2_O, while 5% of valves were set at lower pressures (≤110 mmH_2_O), and 17% at higher pressures (≥140 mmH_2_O). Data regarding initial settings were not available in 12 patients (10.7%). Data regarding the selected management, valve characteristics, and settings are presented in Table 1.

### 3.5. Shunt Revision: Descriptive Analysis

Group B data were used for descriptive statistical purposes but excluded from our analysis regarding shunt failure rates and time to revision. Fourteen patients in Group A undergoing revision were also included for descriptive statistical purposes. Patients who underwent ETV were excluded from all revision-related analyses, although ETV failure was noticed in only one patient, who finally underwent VP shunting. Thus, a total of 37 patients underwent revision, comprising our study population. Notably, of the 37 VP shunt revisions, 8/37 patients (21.6%) required multiple revisions. The median age of these patients was 45 years (IQR = 35). Regarding the etiology of their hydrocephalus, iNPH was the most frequently observed, accounting for nine (24.3%) cases, followed by post-hemorrhagic hydrocephalus in eight (21.6%) cases. Tumor-related hydrocephalus accounted for seven (18.9%) of the revised cases, whereas congenital hydrocephalus accounted for six (16.2%) cases. Furthermore, four (10.8%) post-traumatic and three (8.1%) post-infectious hydrocephalus cases underwent shunt revision.

**Figure 2:**
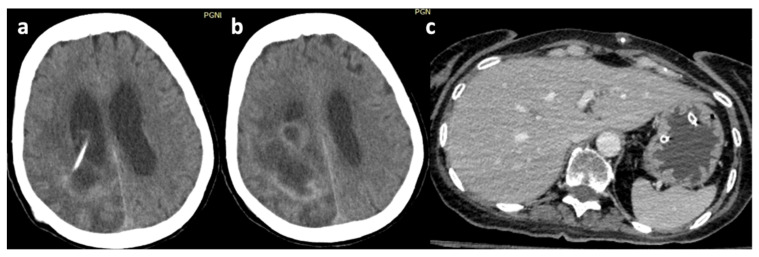
Axial head CT image showing: (**a**). infection of the brain parenchyma adjacent to the ventricular catheter and (**b**). an organized abscess cavity in the brain parenchyma. (**c**). Axial abdominal CT image demonstrating an abdominal abscess surrounding the tip of the peritoneal catheter.

Infection was the most common cause of revision, observed in 16/37 (43.2%) cases (Figure 2). A CSF infection was documented in six cases, while a non-CSF infection was documented in 10. In the CSF infection cases, the most commonly isolated organisms were *Staphylococcus* species (in three patients), *Acinetobacter baumanni* (in one patient), and *Klebsiella* species (in one patient), while no causative organism was identified in one patient. In the non-CSF infection group, *Klebsiella* species were isolated in three patients, and *Acinetobacter baumanni* in two patients. Various *Staphylococcus* species (*aureus*, *haemolyticus*, or *epidermidis*) were also isolated in four patients, while *Candida auris* was isolated in one patient.

**Figure 3:**
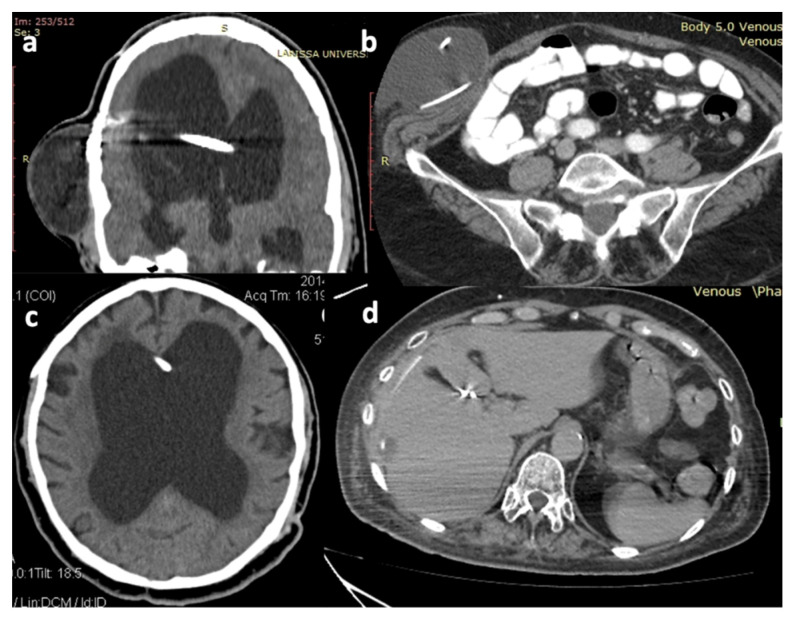
Head and abdominal CT scans demonstrating characteristic cases of VP shunt malfunctions: (**a**). coronal head CT image showing obstruction of the ventricular catheter leading to subcutaneous CSF infusion, (**b**). axial abdominal CT image showing subcutaneous CSF infusion due to migration of the inserted peritoneal catheter into the abdominal wall, (**c**). axial head CT image showing retraction and obstruction of the previously inserted ventricular catheter with enlargement of the ventricular system, and (**d**). axial abdominal CT image showing peripheral migration of the peritoneal catheter into the liver parenchyma.

Obstruction was the second most common cause of shunt failure, observed in six (16.2%) cases. Proximal obstruction was observed in five cases, while in one case, the blockage was peripheral (Figure 3). Moreover, distal catheter migration/extrusion occurred in five (13.5%) patients, whereas proximal catheter migration occurred in another two (5.4%) patients (Figure 3).

**Figure 4:**
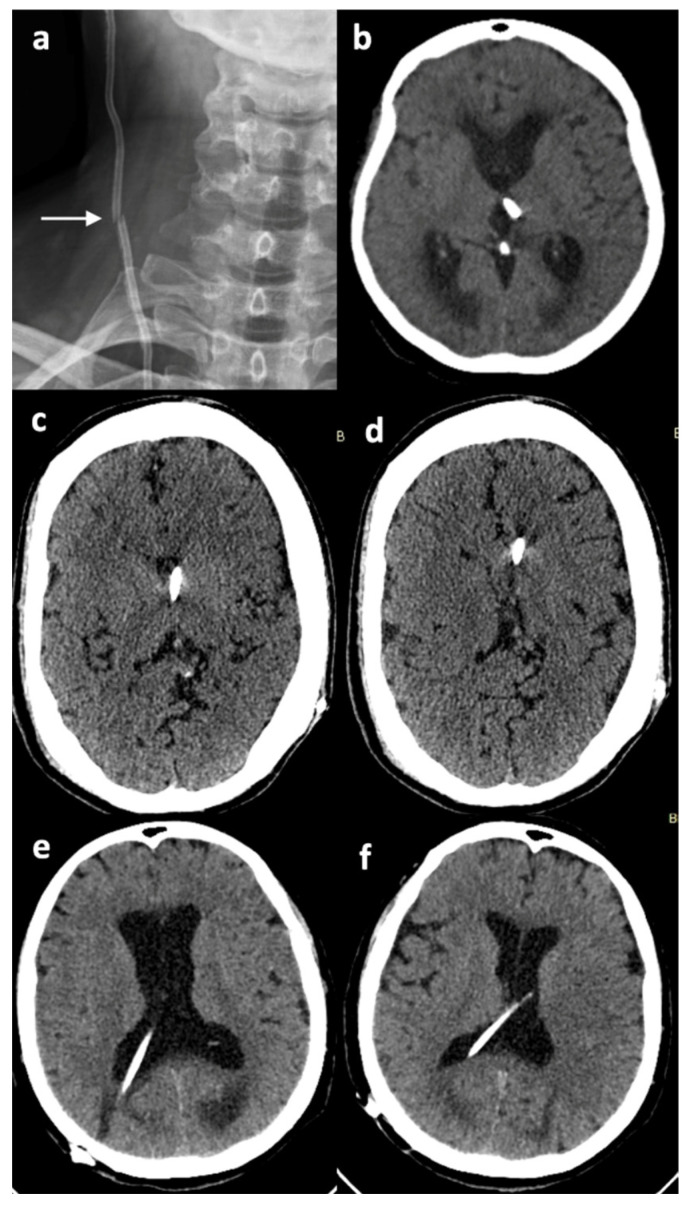
VP shunt malfunctions: (**a**). antero-posterior cervical X-ray showing breakage of the peripheral catheter (arrow), (**b**). axial head CT image showing malposition of the ventricular catheter, (**c**,**d**). slit ventricles due to shunt over-drainage, (**e**). ventricular enlargement due to compromised drainage of the inserted shunt, and (**f**). improvement in ventricular dilatation after revising the valve mechanism and adjusting the opening pressure at a lower setting, on the same patient.

Shunt-associated malfunctions occurred in four (10.8%) cases, whereas mechanical disconnection occurred in two (5.4%) patients (Figure 4). Other causes of revision included lack of communication between the left and right lateral ventricle necessitating the insertion of another shunt on the contralateral side (one patient, 2.7%). Finally, data regarding the etiology of revision was missing in one case. All revision-associated data is summarized in Table 2.

### 3.6. Shunt Failure Rate and Time-to-Revision Analysis

For shunt failure rate and time-to-revision analysis, only Group A data was used. Three patients treated with ETV were excluded from this analysis, resulting in a cohort of 88 patients in total. During 12-month follow-up, 12 patients (13.6%) required surgical revision. Baseline characteristics stratified by revision status are presented in Table 3. Patients who underwent revision were younger (mean age 49.9 ± 24.0 years) compared with those without revision (60.3 ± 13.8 years). Sex distribution and hydrocephalus classification were comparable between groups (Table 3).

#### 3.6.1. Revision-Free Survival

**Figure 5:**
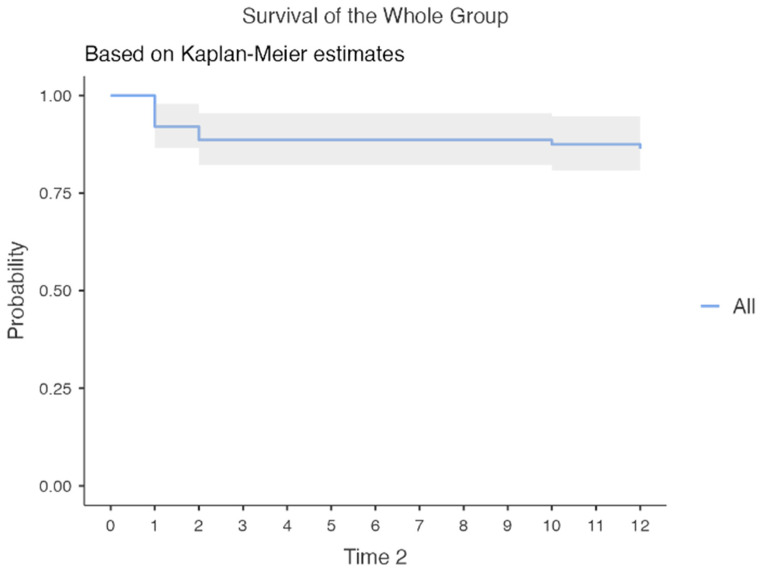
Kaplan–Meier revision-free survival of the entire cohort: Kaplan–Meier curve demonstrating 12-month revision-free survival following ventriculoperitoneal shunt placement in the overall cohort (*n* = 88). Shunt failure was defined as the need for surgical revision. Revision-free survival probabilities were 92.0% at 1 month, 88.6% at 3 months, and 86.4% at 12 months. Median revision-free survival was not reached within the 12-month follow-up period. Shaded areas represent 95% confidence intervals.

Kaplan–Meier analysis demonstrated high revision-free survival throughout follow-up (Figure 5). Survival probabilities were 92.0% at 1 month, 88.6% at 3 months, and 86.4% at 12 months (Table 4). Median revision-free survival was not reached within the study period.

#### 3.6.2. Subgroup Analysis (iNPH vs. Secondary)

**Figure 6:**
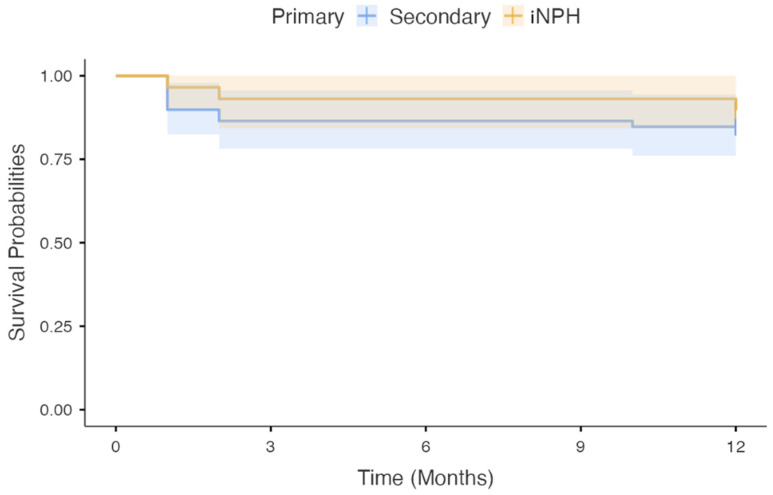
Kaplan–Meier revision-free survival stratified by classification: Kaplan–Meier curves comparing revision-free survival between patients with secondary hydrocephalus (*n* = 59) and idiopathic normal-pressure hydrocephalus (iNPH) (*n* = 29). No significant difference in survival distribution was observed between groups (log-rank test, χ^2^ = 0.50, *p* = 0.479). Median revision-free survival was not reached in either subgroup.

In subgroup analysis (Figure 6), nine cases of revision occurred among 59 patients with secondary hydrocephalus, and three revisions among 29 patients with iNPH. Revision-free survival did not significantly differ between groups (log-rank χ^2^ = 0.50, *p* = 0.479).

#### 3.6.3. Predictors of Revision

**Figure 7:**
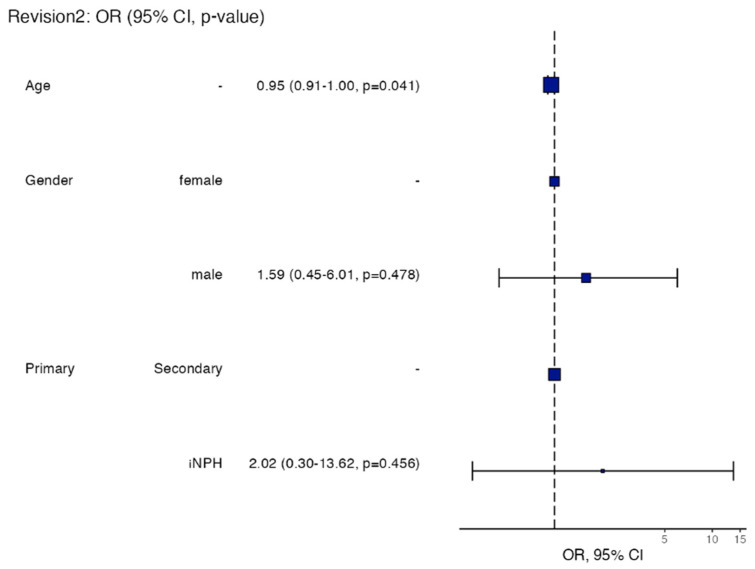
Forest plot of univariable and multivariable logistic regression analyses: forest plot illustrating odds ratios (ORs) with 95% confidence intervals for predictors of 12-month shunt revision. Variables included age (per year increase), sex (male vs. female), and classification (idiopathic NPH vs. secondary). Increasing age was independently associated with lower odds of revision in both univariable and multivariable analyses, whereas sex and classification were not significant predictors. Vertical dashed line indicates null value (OR = 1).

In univariable analysis, increasing age was associated with lower odds of revision (OR 0.96 per year increase; 95% CI 0.93–1.00; *p* = 0.041). Sex and classification were not significantly associated with revision. In multivariable analysis adjusting for age, sex, and classification, age remained independently associated with revision (adjusted OR 0.95 per year increase; 95% CI 0.91–1.00; *p* = 0.041). Neither male sex (adjusted OR 1.59; 95% CI 0.45–6.01; *p* = 0.478) nor iNPH classification (adjusted OR 2.02; 95% CI 0.30–13.62; *p* = 0.456) was a significant predictor. Model discrimination was modest (c-statistic = 0.617), and calibration was acceptable (Hosmer–Lemeshow *p* = 0.143). Univariable and multivariable logistic regression analyses are presented in Table 5, as well as in Figure 7 in the form of a forest plot.

#### 3.6.4. Nomogram

**Figure 8:**
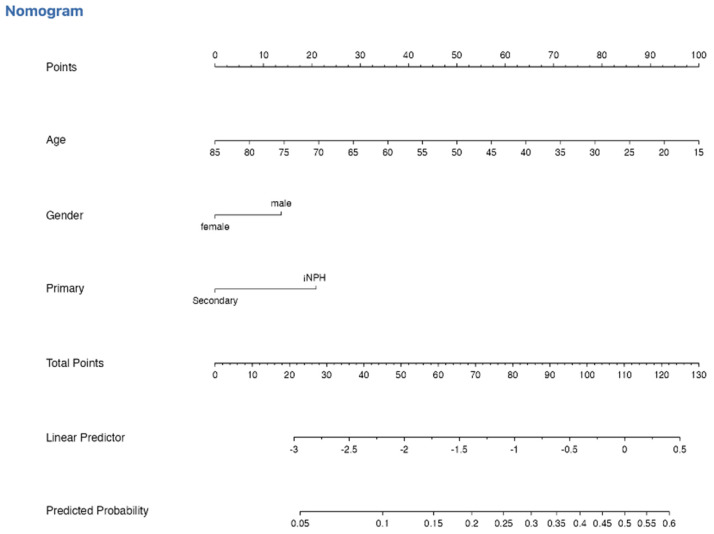
Nomogram for prediction of 12-month shunt revision derived from the multivariable logistic regression model for estimating individual probability of shunt revision within 12 months. Points are assigned for each predictor (age, sex, and classification), summed to generate a total score corresponding to predicted revision probability. Model discrimination was modest (c-statistic = 0.617), with acceptable calibration (Hosmer–Lemeshow *p* = 0.143).

A nomogram was constructed from the multivariable model to estimate the individualized probability of 12-month revision (Figure 8). The graphical tool assigns weighted points to age, sex, and classification, with total points corresponding to predicted risk. It is used by locating the patient’s value for each predictor (age, sex, and hydrocephalus classification) on the corresponding axis and projecting vertically to the point scale to assign a score. The individual scores are then summed to obtain a total point value, which is subsequently projected downward to the probability scale to estimate the predicted risk of ventriculoperitoneal shunt revision within 12 months. Higher total points correspond to a higher estimated probability of revision. The nomogram is intended to assist in individualized risk stratification and clinical counseling and should be interpreted in conjunction with overall clinical assessment.

## 4. Discussion

Our study provides common clinical practice data based on a single-center hydrocephalus registry for adults. Data regarding the etiology, treatment decision-making, shunt failure, and revision rate in patients with hydrocephalus was retrospectively collected and presented. In regard to the underlying etiology, iNPH (30.7%), post-hemorrhagic (23.7%), and tumor-related (21.1%) were the most commonly observed causes. Less common types of hydrocephalus, such as post-infectious and post-traumatic, were also observed. Additionally, congenital hydrocephalus, manifesting in adulthood, was present in a limited number of patients.

In this single-center cohort of 88 patients undergoing ventriculoperitoneal shunt diversion, we observed a 12-month revision rate of 13.6%, with overall revision-free survival of 86.4% at one year. Most failures occurred early during follow-up, and median revision-free survival was not reached within 12 months. Survival analysis demonstrated no significant difference in revision rate between patients with iNPH and those with secondary hydrocephalus. In the regression analysis, increasing age was independently associated with lower odds of shunt revision, whereas sex and hydrocephalus classification were not significant predictors. Based on the multivariable model, we developed a nomogram to provide individualized risk estimation, although model discrimination was modest.

Data from previously published studies regarding the etiology of hydrocephalus demonstrate significant variation. Overall, the most common causes of adult hydrocephalus are iNPH, which accounts for approximately 30% of cases, and tumor-related hydrocephalus, responsible for 20–36% [14,15,16]. Post-hemorrhagic hydrocephalus accounts for another 13–25% of cases [14,15,16]. The previously reported percentages are in agreement with our findings. A recently published meta-analysis by Isaacs et al. [13] found that iNPH was the most common cause of adult hydrocephalus, at an approximate rate of 16%, followed by post-hemorrhagic (12.6%) and tumor-related hydrocephalus (12.6%) [13]. These findings are in agreement with our results regarding the relative frequency of causes, although the reported incidences are lower [13]. Interestingly, they reported that the median age of hydrocephalus patients requiring CSF diversion surgery ranged from 58 to 60 years old [13], which is almost identical to our current findings (median age: 59 years). On the other hand, Bir et al. reported that vascular pathology was the most common, followed by tumor-associated hydrocephalus, while iNPH was identified as the third most common cause in their series [4]. This difference may be explained by the multiracial consistency of their study population [4]. Indeed, our single-center study included exclusively patients of Caucasian origin identifying iNPH as the most common cause. It has been reported that iNPH is significantly higher among Caucasian patients [4]. It also needs to be pointed out that the prevalence of iNPH increases as patient age advances; therefore, adult populations, shifted towards the seventh or eighth decade of life, have the tendency to demonstrate higher prevalence rates [20].

The overall shunt failure rate in our cohort was 15.9%. Khalil et al. reported in their retrospective series a similar failure rate of 20.1% [17]. Isaacs et al. found that the overall shunt failure rate in series with one year follow-up was 10%, and that in studies with follow-up between one and two years was 12%, while this rate became approximately 32% at two years [13]. Anderson et al. reported an overall 30-day failure rate of 21.5% for adults, while this percentage increased to 34.8% at one year [20]. It has previously been reported that the failure rate of a newly inserted VP shunt exceeds 20% at 12-month follow-up [21,22]. A meta-analysis, including 46 articles and 13,603 shunt insertions, found an overall shunt failure rate of 19.2% [23]. The authors also reported that there was a significant decreasing failure rate of approximately 2% per year [23]. Likewise, Merkler et al. found in their retrospective study that most complications clustered in the first year after a VP shunt insertion [24]. Similarly, we found that in our newly inserted shunts, the median time for revision was 27.5 days. Khan et al. reported an overall failure rate of 15.4% within 120 days [25].

Furthermore, the stratification of our failure rate data according to the cause of hydrocephalus showed a 22.7% failure rate in post-hemorrhagic cases, followed by tumor-related cases (21.7%) and iNPH (10.3%). Likewise, Anderson et al. found that etiology of hydrocephalus was associated with failure [20]. They demonstrated that post-infectious hydrocephalus had the highest failure rate, followed by tumor-associated hydrocephalus, idiopathic intracranial hypertension, and post-traumatic hydrocephalus [20]. According to Khan et al., the most common etiologies of hydrocephalus with malfunction were postcranial surgery in 13.2%, tumor-related in 17.3%, and iNPH in 8.8% [25]. In the same study, post-hemorrhagic hydrocephalus was responsible for failure in 3.2% of cases [25].

In regard to the cause of shunt failure, we found that infection was the most common cause, observed in 43.2% of our cases, followed by obstruction in 16.2%, and shunt malfunction in 10.8%. Isaacs et al. identified obstruction as the main cause of shunt failure, occurring in 23.2%, and infection as the second most common cause, occurring in 22.5% [13]. Paff et al. reported similar results [18]. Khalil et al. also found that the most common cause of shunt failure was infection, occurring in 24% of cases [17]. Machaku et al. found that infection was the most common cause (50%), followed by malposition (27.8%) and obstruction (15.6%) [26].

In our cohort, proximal and distal failures (due to catheter migration or obstruction) occurred at similar rates. However, Isaacs et al. reported that the most common location of shunt failure was the distal catheter, in 33.4% of cases, whereas proximal failure accounted for 21.6% [13]. The proximal catheter is the most common site of obstruction in pediatric populations [27,28,29]. Kast et al. have postulated that the proximal catheter is a more common cause of failure in early failures, while distal obstruction/malfunction occurs more frequently among delayed failures [19]. Interestingly, Desai et al. found that when comparing fixed vs. programmable valves, programmable valves had a significantly lower malfunction rate as well as complication rate within 180 days [30]. Similarly, Stewart et al. reported that when comparing programmable vs. non-programmable shunts, the use of a programmable one was associated with a 46% decrease in the risk of failure [16]. Likewise, Lee et al. found that the usage of programmable shunts was associated with lower failure and revision rates in adults [31]. However, Katiyar et al. found in their study that programmable shunts demonstrated no advantage over non-programmable shunts in iNPH patients [32]. Chen et al. reported that the usage of programmable shunts may be advantageous for patients with specific hydrocephalus etiologies, such as iNPH and post-traumatic hydrocephalus [33].

One of the findings of our current study was that increased patient age was significantly associated with a lower risk of shunt failure. This finding, however, should be interpreted with caution. Our study included only 14 shunt failure cases. Therefore, a multivariate analysis could not be performed. Thus, the extrapolation of our findings to general adult hydrocephalus patients may carry significant risks. Another recent retrospective cohort study [17] showed that younger age predicted early shunt failure, whereas an older one reported opposite results [25]. Interestingly, a similar study in adults showed that younger age was also associated with higher risk of revision in a binary univariate analysis; however, this was not the case in multivariate analysis models [34]. It also has to be taken into consideration that comorbidities among elderly patients may play a role in the development of any shunt-related complications.

### Study Limitations

This study has several limitations that need to be acknowledged. Firstly, the relatively small sample size and the limited number of revision events (*n* = 12) may reduce statistical power and increase the risk of model instability. Although multivariable analysis was performed, the low events-per-variable ratio raises the possibility of overfitting, and the predictive performance of the nomogram should therefore be cautiously interpreted, for patient consultation but not for decision-making. Needless to say, further larger-sample studies are required to validate our findings. Secondly, the follow-up period was limited to 12 months. While most shunt failures occur early, late failures may not have been captured, thus potentially underestimating long-term revision risk. Thirdly, due to the retrospective nature of our study, only a limited number of clinical variables were included in the regression model. Other potentially relevant factors—such as shunt type, valve settings, etiology-specific characteristics, perioperative complications, and comorbidities—were not incorporated and might influence revision risk. Finally, although model calibration was acceptable, discrimination was modest, indicating limited ability to distinguish between patients who will and will not require revision. Therefore, the nomogram should be considered a supportive risk-stratification tool rather than a definitive predictive instrument.

## 5. Conclusions

In our current retrospective cohort of patients undergoing shunt placement, the revision-free survival at 12 months was high, with most failures occurring early after surgery. Revision rates did not significantly differ between iNPH cases and cases with secondary hydrocephalus. It seems that increasing age is independently associated with a lower likelihood of revision, whereas sex and hydrocephalus classification are not significant predictors. A nomogram derived from the multivariable model could provide an individualized estimate of revision risk; however, given the modest discriminative performance and limited number of events, it should be considered a supportive risk-stratification tool. External validation in larger, multicenter cohorts is warranted before routine clinical implementation.

## Figures and Tables

**Table 1:** Demographics, hydrocephalus etiology, area of obstruction, management, and valve characteristics and pressure settings of our cohort.

Characteristic	*n* (%)
Patients	114
Median age (years, IQR)	59, 26.5
Sex	
Male	58 (50.9%)
Female	56 (49.1%)
Male-to-female ratio	1.04:1
**Etiology**	
Idiopathic normal-pressure hydrocephalus	35 (30.7%)
Secondary hydrocephalus	79 (69.3%)
Post-hemorrhagic	27 (23.7%)
Tumor-related	24 (21.1%)
Post-traumatic	10 (8.8%)
Congenital	8 (7%)
Post-infectious	6 (5.3%)
Other	4 (3.5%)
**Area of obstruction**	
Foramen of Monroe	5 (4.4%)
3rd ventricle	4 (3.5%)
Cerebral aqueduct	15 (13.2%)
4th ventricle	6 (5.3%)
Subarachnoid space	83 (72.8%)
Unknown	1 (0.9%)
**Management option**	
VP shunt	110 (96.5%)
VA shunt	1 (0.9%)
ETV	3 (2.6%)
**Shunt type (total number of shunts inserted: 112)**	
Codman Hakim	102 (91.1%)
Integra OSV II	1 (0.9%)
Polaris	2 (1.7%)
Sphera Pro	2 (1.7%)
Unknown	5 (4.5%)
**Valve setting (mmH_2_O) (total number of shunts inserted: 112)**	
Low (≤110)	5 (4.5%)
Medium (120–130)	78 (69.6%)
High (≥140)	17 (15.2%)
Unknown	12 (10.7%)

IQR, interquartile range; VP, ventriculoperitoneal; VA, ventriculoatrial; ETV, endoscopic third ventriculostomy.

**Table 2:** Distribution of shunt failure etiology.

Failure Etiology	Patients (*n*)	Revision Rate (%)
1. Infection	16	43.2%
Non-CSF	10	
CSF	6	
2. Obstruction	6	16.2%
Proximal	5	
Distal	1	
3. Migration	7	18.9%
Proximal	2	
Distal	5	
4. Valve malfunction	4	10.8%
5. Disconnection	2	5.4%
6. Other	1	2.7%
7. No data	1	2.7%

CSF, cerebrospinal fluid.

**Table 3:** Baseline characteristics according to 12-month shunt revision rate.

Variable	No Revision (*n* = 76)	Revision (*n* = 12)	*p*-Value
Age, years, mean (SD)	60.3 (13.8)	49.9 (24.0)	0.041 *
Sex, *n* (%)			0.482
Female	40 (52.6)	5 (41.7)	
Male	36 (47.4)	7 (58.3)	
Classification, *n* (%)			0.531
Secondary	50 (65.8)	9 (75.0)	
Idiopathic normal-pressure hydrocephalus (iNPH)	26 (34.2)	3 (25.0)	

* Statistically significant.

**Table 4:** Kaplan–Meier revision-free survival analysis.

**A. Overall Revision-Free Survival (*n* = 88)**				
**Time (Months)**	**Number at Risk**	**Events**	**Survival Probability (%)**	**95% CI**
1	88	7	92.0	86.6–97.9
3	78	3	88.6	82.2–95.5
6	78	0	88.6	82.2–95.5
9	78	0	88.6	82.2–95.5
12	77	2	86.4	79.5–93.8
**B. Subgroup Analysis by Classification**				
**Classification**	**N**	**Observed Events**	**Censored**	**Expected Events**
Secondary	59	9	50	7.96
Idiopathic normal-pressure hydrocephalus (iNPH)	29	3	26	4.04

Total revisions: 12/88 (13.6%). Median revision-free survival: not reached within 12 months. Log-rank test: χ^2^ = 0.50; df = 1; *p* = 0.479.

**Table 5:** Univariable and multivariable logistic regression for 12-month shunt revision.

Variable	Univariable OR (95% CI)	*p*-Value	Multivariable OR (95% CI)	*p*-Value
Age (per year increase)	0.96 (0.93–1.00)	0.041	0.95 (0.91–1.00)	0.041
Male vs. female	1.56 (0.46–5.67)	0.482	1.59 (0.45–6.01)	0.478
Idiopathic NPH vs. secondary	0.64 (0.13–2.36)	0.531	2.02 (0.30–13.62)	0.456

Model performance: AIC = 72.9; C-statistic = 0.617; Hosmer–Lemeshow χ^2^(8) = 12.19, *p* = 0.143.

## Data Availability

The data presented in this study are available on request from the corresponding author due to ethical reasons.

## Abbreviations

The following abbreviations are used in this manuscript:

AVM	Arteriovenous malformation
CI	Confidence interval
CM	Cavernous malformation
CNS	Central nervous system
CSF	Cerebrospinal fluid
ETV	Endoscopic third ventriculostomy
HIPAA	Health Insurance Portability and Accountability Act
iNPH	Idiopathic normal-pressure hydrocephalus
IQR	Interquartile range
LP	Lumboperitoneal
OR	Odds ratio
SAH	Subarachnoid hemorrhage
VAS	Ventriculoatrial shunt
VPS	Ventriculoperitoneal shunt
